# Social Exclusion and Material Disadvantage: Housing, Poverty, and Living Standards Impacts

**DOI:** 10.1093/geroni/igaa057.2506

**Published:** 2020-12-16

**Authors:** Charles Waldegrave, Chris Phillipson

## Abstract

SRPP 2020 Ollie Randall Symposium Award Winner. Many of the detrimental effects of material disadvantage on the lives of citizens have been well understood by public health and social scientists, and post-World War II social protection polices were designed to mitigate the negative impacts of them. As the numbers of older people increase proportionally to the rest of the population in most countries, less is known about the exclusionary impacts of material disadvantage and the roles housing, poverty and living standards play on the health, well-being and social connections of their lives. This symposium draws together research emanating out of four countries Norway, Poland, Ireland and New Zealand that are part of the European COST Action 15122 Reducing Old-Age Social Exclusion: Collaborations in Research and Policy (ROSEnet). The papers present contemporary results of specific health well-being and social impacts of material disadvantage in the four quite different countries and assesses them through the lens of social exclusion. As the growing international evidence during the last decade has highlighted the negative health and well-being impacts of loneliness and social disconnection (Holt-Lunstad et. al. 2015), the role of housing, poverty and living standards has in creating social exclusion is less well known. This research analyses the subjective and objective experiences of material disadvantage and quantifies their exclusionary impacts on well-being (e.g. quality of life, loneliness), health functioning (mental and physical) and their challenges to macro-structures (e.g. government policies, social protection).

